# Supplementary material for: Risk factors for third-generation cephalosporin resistant Enterobacteriaceae in gestational urine cultures: A retrospective cohort study based on centralized electronic health records
Source: PLoS One. 2020 Jan 3;15(1):e0226515. doi: 10.1371/journal.pone.0226515 (PMC6941821; doi:10.1371/journal.pone.0226515)
Supplement: S1 File — (DOCX) [file pone.0226515.s001.docx]

STROBE Statement—checklist of items that should be included in reports of observational studies

|  | Item No. | Recommendation | Page  No. | Relevant text from manuscript |
| --- | --- | --- | --- | --- |
| **Title and abstract** | 1 | (*a*) Indicate the study’s design with a commonly used term in the title or the abstract | 1 | **Risk factors for third-generation cephalosporin resistant *Enterobacteriaceae* in gestational urine cultures: A retrospective cohort study based on centralized electronic health records.** |
|  |  | (*b*) Provide in the abstract an informative and balanced summary of what was done and what was found | 2 | Third-generation-cephalosporin resistant *Enterobacteriaceae* (3GCR-EB) carriage in pregnant women poses challenges for infection control and therapeutic decisions. The factors associated with multidrug resistant *Enterobacteriaceae* carriage in the gestational period are not well documented…. |
| Introduction | | | |  |
| Background/rationale | 2 | Explain the scientific background and rationale for the investigation being reported | 4 | *Enterobacteriaceae* (EB) species are a well-established cause of obstetric and neonatal infections …. |
| Objectives | 3 | State specific objectives, including any prespecified hypotheses | 5 | We aimed to identify risk factors associated with 3GC-resistant carbapenem sensitive EB isolation in urine of pregnant women… |
| Methods | | | |  |
| Study design | 4 | Present key elements of study design early in the paper | 6 | A retrospective cohort study of women who delivered … |
| Setting | 5 | Describe the setting, locations, and relevant dates, including periods of recruitment, exposure, follow-up, and data collection | 6 | CHS is Israel’s largest integrated payer-provider healthcare system… |
| Participants | 6 | (*a*) *Cohort study*—Give the eligibility criteria, and the sources and methods of selection of participants. Describe methods of follow-up  *Case-control study*—Give the eligibility criteria, and the sources and methods of case ascertainment and control selection. Give the rationale for the choice of cases and controls  *Cross-sectional study*—Give the eligibility criteria, and the sources and methods of selection of participants | 6 | retrospective cohort study of women who delivered in eight Israeli hospitals of the Clalit Health Services (CHS) system between 2009 and 2013 and had performed at least one urine culture within one year prior to delivery with at least one positive urine culture for EB. |
|  |  | (*b*) *Cohort study*—For matched studies, give matching criteria and number of exposed and unexposed  *Case-control study*—For matched studies, give matching criteria and the number of controls per case |  |  |
| Variables | 7 | Clearly define all outcomes, exposures, predictors, potential confounders, and effect modifiers. Give diagnostic criteria, if applicable | 8 | The main outcome was isolation of 3GCR-EB in gestational urine culture. … |
| Data sources/ measurement | 8* | For each variable of interest, give sources of data and details of methods of assessment (measurement). Describe comparability of assessment methods if there is more than one group | 7,8 | Data collection. |
| Bias | 9 | Describe any efforts to address potential sources of bias | 9 | A multivariate analysis using the Generalized Estimating Equations (GEE) model… |
| Study size | 10 | Explain how the study size was arrived at | 5 | All women who delivered… |

Continued on next page

| Quantitative variables | 11 | Explain how quantitative variables were handled in the analyses. If applicable, describe which groupings were chosen and why |  | NA |
| --- | --- | --- | --- | --- |
| Statistical methods | 12 | (*a*) Describe all statistical methods, including those used to control for confounding | 9 | Descriptive statistics were calculated for all variables … |
|  |  | (*b*) Describe any methods used to examine subgroups and interactions | 9 | A multivariate analysis using the Generalized Estimating Equations… |
|  |  | (*c*) Explain how missing data were addressed |  | NA |
|  |  | (*d*) *Cohort study*—If applicable, explain how loss to follow-up was addressed  *Case-control study*—If applicable, explain how matching of cases and controls was addressed  *Cross-sectional study*—If applicable, describe analytical methods taking account of sampling strategy | Not applicable |  |
|  |  | (*e*) Describe any sensitivity analyses |  |  |
| Results | | | | |
| Participants | 13* | (a) Report numbers of individuals at each stage of study—eg numbers potentially eligible, examined for eligibility, confirmed eligible, included in the study, completing follow-up, and analysed | 10, fig 1 | The study included 134,152 women who had urine…, |
|  |  | (b) Give reasons for non-participation at each stage | Fig 1 | Of which... |
|  |  | (c) Consider use of a flow diagram | Fig 1 |  |
| Descriptive data | 14* | (a) Give characteristics of study participants (eg demographic, clinical, social) and information on exposures and potential confounders | 10 | Most women in our cohort… |
|  |  | (b) Indicate number of participants with missing data for each variable of interest | irrelevant |  |
|  |  | (c) *Cohort study*—Summarise follow-up time (eg, average and total amount) | 5 |  |
| Outcome data | 15* | *Cohort study*—Report numbers of outcome events or summary measures over time | 12 | As shown in table 1… |
|  |  | *Case-control study—*Report numbers in each exposure category, or summary measures of exposure |  |  |
|  |  | *Cross-sectional study—*Report numbers of outcome events or summary measures |  |  |
| Main results | 16 | (*a*) Give unadjusted estimates and, if applicable, confounder-adjusted estimates and their precision (eg, 95% confidence interval). Make clear which confounders were adjusted for and why they were included | 13 | Several variables in the univariate analysis were significantly… |
|  |  | (*b*) Report category boundaries when continuous variables were categorized |  |  |
|  |  | (*c*) If relevant, consider translating estimates of relative risk into absolute risk for a meaningful time period | irrelevant |  |

Continued on next page

| Other analyses | 17 | Report other analyses done—eg analyses of subgroups and interactions, and sensitivity analyses | 13 | Using the GEE.. |
| --- | --- | --- | --- | --- |
| Discussion | | | | |
| Key results | 18 | Summarise key results with reference to study objectives | 14 | This study identified.. |
| Limitations | 19 | Discuss limitations of the study, taking into account sources of potential bias or imprecision. Discuss both direction and magnitude of any potential bias | 17 | This study has several limitations. First, due |
| Interpretation | 20 | Give a cautious overall interpretation of results considering objectives, limitations, multiplicity of analyses, results from similar studies, and other relevant evidence | 18 | Information of previous antibiotic treatments and hospitalization in the last year and AFPs are easily accessed and can be used for appropriate infection control practices and antimicrobial therapy |
| Generalisability | 21 | Discuss the generalisability (external validity) of the study results | 18 | However, with the growing use of EHRs, the relevant data from community clinics may be increasingly available in many inpatient healthcare facilities. |
| Other information | |  | | |
| Funding | 22 | Give the source of funding and the role of the funders for the present study and, if applicable, for the original study on which the present article is based | NA |  |

*Give information separately for cases and controls in case-control studies and, if applicable, for exposed and unexposed groups in cohort and cross-sectional studies.

**Note:** An Explanation and Elaboration article discusses each checklist item and gives methodological background and published examples of transparent reporting. The STROBE checklist is best used in conjunction with this article (freely available on the Web sites of PLoS Medicine at http://www.plosmedicine.org/, Annals of Internal Medicine at http://www.annals.org/, and Epidemiology at http://www.epidem.com/). Information on the STROBE Initiative is available at www.strobe-statement.org.
